# More support for mothers: a qualitative study on factors affecting immunisation behaviour in Kampala, Uganda

**DOI:** 10.1186/1471-2458-11-723

**Published:** 2011-09-25

**Authors:** Juliet N Babirye, Elizeus Rutebemberwa, Juliet Kiguli, Henry Wamani, Fred Nuwaha, Ingunn MS Engebretsen

**Affiliations:** 1 School of Public Health, Makerere University College of Health Sciences, P.O. Box 7072, Kampala Uganda; 2 Centre for International Health, University of Bergen, Bergen, Norway

## Abstract

**Background:**

The proportion of Ugandan children who are fully vaccinated has varied over the years. Understanding vaccination behaviour is important for the success of the immunisation programme. This study examined influences on immunisation behaviour using the attitude-social influence-self efficacy model.

**Methods:**

We conducted nine focus group discussions (FGDs) with mothers and fathers. Eight key informant interviews (KIIs) were held with those in charge of community mobilisation for immunisation, fathers and mothers. Data was analysed using content analysis.

**Results:**

Influences on the mother's immunisation behaviour ranged from the non-supportive role of male partners sometimes resulting into intimate partner violence, lack of presentable clothing which made mothers vulnerable to bullying, inconvenient schedules and time constraints, to suspicion against immunisation such as vaccines cause physical disability and/or death.

**Conclusions:**

Immunisation programmes should position themselves to address social contexts. A community programme that empowers women economically and helps men recognise the role of women in decision making for child health is needed. Increasing male involvement and knowledge of immunisation concepts among caretakers could improve immunisation.

## Background

Each year, over 24 million children under one year of age miss routine immunisation services. Seventy percent of these children live in ten countries including Uganda [1]. Several studies identify demographic characteristics of the caretakers with suboptimal utilisation of immunisation services [2-9]. Others have identified health system factors and behavioural influences on immunisation [10-12]. Those studies on behavioural factors have been done in settings with almost universal access to immunisation services.

In low income settings, immunisation programmes have traditionally targeted women and neglected the role of men. Power relations within the household and between kin and friends affect health decision making [13]. Often the socio-cultural context relevant for health seeking behaviour is not considered during programme implementation.

Immunisation outreaches have been tailored to rural areas, where access to health services is low due to poor road network. The rationale for this being the majority of the population live in rural areas. A growing concern with rapid urbanisation is how to provide immunisation services. This study examined influences on childhood immunisation behaviour using the attitude-social influence-self efficacy model.

## Attitude-social influence-self efficacy model

**Figure 1:**
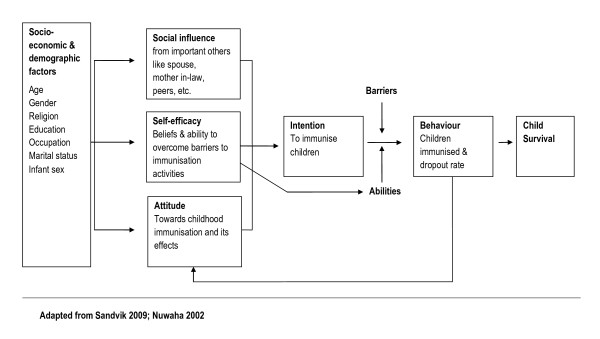
**Attitude-social influence-self efficacy model**.

The attitude-social influence-self efficacy model (shown in figure 1) was originally developed by de Vries et al for smoking cessation [14]. In the model presented in this paper behaviour related to childhood immunisation is the result of behaviour intention. This intention is in turn predicted by three main factors: social influence, self efficacy, and attitude [14-16]. A person's attitude refers to the extent to which a person has a favourable or unfavourable evaluation of the behaviour. A person's attitude towards childhood immunisation may be influenced by personal beliefs such as misconceptions associated with immunisation of children, and by the fear associated with side effects from vaccines. This fear is a barrier to optimal utilization of immunisation services.

Social influence results from social norms related to immunisation of children and the support from important others like the partner or the mother. Self efficacy refers to a person's ability to cope with barriers that may hinder adherence to recommended immunisation schedules. A low perceived benefit of immunisation would reduce the ability to cope with the barriers to immunisation services. Self efficacy not only influences behaviour intention but also directly influences behaviour. Barriers and abilities could influence behaviour related to immunisation activities. Previous behaviour or trying to perform the behaviour has a feedback mechanism that in turn influences the attitude, social influence and self efficacy.

Thus this model infers that attitude, social influence, and self efficacy variables can be targeted through health promoting activities for improving immunisation coverage in addition to reducing delay in immunisations, whereas external variables like socio-economic and demographic variables are usually not easily changeable. On the other hand, the demographic features would be valuable in identifying individuals who fail to complete immunisation schedules or do not immunise their children [15-17].

## Methods

### Study area

The study was conducted in Kampala from June to September 2010. Kampala is the largest urban area and capital city of Uganda with an average population density of more than 7400 persons per square kilometre and a total population of about 1.6 million people. Children below 5 years constitute 20% of the total population. Immunisation coverage from surveys in Kampala show BCG coverage at 91%, combined pertusis vaccine at 68%, Polio3 at 56%, measles at 71% and those who receive all expanded immunisation programme (EPI) vaccines at 47% [18,19]. These rates are all below the national targets.

Health services in Kampala are provided by government, non-governmental organization (NGO), and privately owned health facilities. All government and NGO facilities provide routine immunisation services in addition to outreach immunisation services.

There are five divisions in Kampala and each division is administratively semi autonomous with a separate work plan and budget. Three of the divisions, Central, Kawempe, and Rubaga, are better served with public health facilities. Nakawa and Makindye divisions are relatively least served by public health facilities and since most immunisation services are provided by public health facilities Nakawa and Makindye divisions are in great need for improved services. This study was conducted in Nakawa and Makindye divisions of Kampala.

### Data collection and study population

The main data collection methods included focus group discussions (FGDs) and key informant interviews (KII).

FGDs which are described as appropriate arenas for discussing and airing multiple views on an issue were chosen as one of the data collection methods [20]. Nine FGDs were conducted with three different categories of respondents; three with mothers aged 18-25 years (referred to as 'FGD with younger mothers'), four with mothers older than 25 years (referred to as 'FGD with older mothers') and two with fathers (referred to as 'FGD with fathers'). Older women were chosen because they play a significant role in society and are looked up to by younger parents for advice. FGDs of fathers were conducted because fathers also play a significant role in childhood immunisation, especially when costs are to be incurred [13,21,22]. With the assumption that men and women tell different stories, and that adults in different age groups feel more like peers within their respective age group, different FGDs were constituted to give evaluation of consensus and contestation of information [20,23]. The local council leaders mobilised mothers and fathers that had children younger than five years, older women and men. Local council leaders are village heads and lead a population of approximately 1000 adults. The entire executive at the village level are nominated and voted into these positions by the adults that constitute a village. Our study team explained the inclusion criteria to the local council leader and to the secretary for women on the village committee. A later date was set for the FGD. Either the local council chairman (for male FGDs) or the secretary for women (for female FGDs) chose one participant per household. The households from which FGD participants were selected were geographically scattered across the village and not clustered in same locality. Those mobilising participants were encouraged to bring up to 25 eligible participants. On the day of the FGD, the study team together with the local leaders or secretary for women then chose individuals who would participate in the discussion. FGDs were conducted in one of the homes of the FGD participants (five FGDs) or inside the community centres (four FGDs). There were a total of 73 participants for FGDs; 58 were women. Each FGD had between six to eleven participants per group discussion.

All discussions lasted 1-2 hours and were conducted in Luganda (local language) except for one FGD that used English as the medium of communication and one FGD that used a Luo speaking translator. The interviews were moderated by one female social scientist and note taking was by one male educated in health promotion. Both had experience in conducting FGDs, and were both fluent in Luganda and English languages. JNB (first author) made observations at all discussions and asked questions to clarify some of the issues raised in the FGDs.

Eight key informant interviews (KIIs) were conducted in the local language, Luganda. Two of the KIIs were held with those in charge of community mobilisation for immunisation, three with fathers and three with mothers in the community. The KIIs were conducted by an experienced research assistant (health promotion). Data was reviewed by JNB and the research assistant after each interview to assess how the questions were being answered before conducting the next KII.

JNB was the principal investigator with medical background and fluent in English and Luganda. She was trained in qualitative methods and had prior experience with qualitative field work in other health related fields. She discussed the experiences of the interviewer and note taker and whether questions were being understood by FGD participants. The FGDs and KIIs were conducted using a guide that focused on beliefs, perceptions, experiences, actions and consequences from immunisation activities. We asked questions such as; the reasons why participants took their children for immunisation, what barriers they faced during the process, and how they overcame these barriers. Reasons for refusal of immunisation were also explored.

The numbers of FGDs/KIIs were deemed sufficient when additional interviews yielded little new information on the core study objectives.

### Data analysis

All the data were tape recorded after obtaining participants' consent. The audio data was transcribed and later translated from the local language into English by the moderators while for two FGDs (one conducted in English and another that used a translator) were transcribed directly into English. Audio records and local language terminologies were kept for consistence checks and compiled with field notes. The researchers listened to the audio recordings to confirm the information on the transcripts. Audio recording enabled details of the KIIs and FGDs to be obtained with accuracy that would not be got from the field notes or from memory alone. Tape recording also allowed more eye contact between the moderators and the respondents [24]. The unit of analysis was the transcripts from FGDs and KIIs. The authors JNB, ER, JK and IMSE read through the transcripts and came up with meaning units individually. They harmonised their meaning units and went back and separately coded the meaning units. These codes were again shared between the four authors. During the discussion, the codes were merged into categories and subsequently into themes which were shared with the other co-authors [25]. The themes were grouped and presented according to the attitude, social influence and self efficacy model. The different data sources informed each other during design, implementation and analysis, thus the data were triangulated during the entire research process [26].

### Ethics approval

Ethics approval was obtained from Makerere University School of Public Health Higher Degrees Research and Ethics Committee (IRB00005876FWA/Protocol 085) and independently from the Uganda National Council for Science and Technology (HS 786). The interviews were conducted only after informed consent was obtained from the study participants.

## Results

The major findings of this study were that; convictions of the caretakers, self efficacy, and the supportive or non-supportive role of significant others influenced the involvement or non-involvement of parents in childhood immunisation (see table 1). In the next section we present two main sub-themes under social influence namely; influence from the male partner and influence from the older generation or peers on immunisation behaviour. The theme on self efficacy presents data on barriers faced during utilisation of immunisation services and the participants' ability to overcome these namely; barriers associated with access to immunisation services and those that arose from personal challenges. Attitudinal factors are categorised into three main sub-themes: trust in immunisation, fear of side effects, and programmatic preferences.

**Table 1:** Emerging themes-direction of influence on immunisation behaviour

Themes	Data source (FGDs, KIIs)	Influence on utilisation of immunisation services
Social influence		
***Influence from male partner***		
Father's offer support in form of transport to immunisation unit	All	Positive
Father's make the decision to immunise	Female FGDs	Both positive and negative
Joint decision to immunise or not	All (emphasized more among male FGDs)	Positive
Less power for decision making	Female FGDs	Negative influence for women
***Influence from older generation and peers***		
Decision making involves kin	All	Mostly negative
Decision to immunise against spousal consent encouraged by experienced mothers	Female FGDs (older women) and female key informant	Positive
Intimate partner violence	Female FGDs	Negative
Social stigma against teenage mothers	Female KII	Negative
		
Self-efficacy		
Difficult to access immunisation unit	All	Negative
Vaccines sometimes out of stock	All	Negative
Have to choose between money for transport and food	All FGDs	Negative
Lack of presentable clothing	Female FGDs	Negative
Gender roles	All	Negative for the men
Lack of job security leading to choice between work and immunisation	All	Negative
		
		
Attitude		
		
***Trust in immunisation***		
Believe immunisation is beneficial	All	Positive
		
Perception of lack of trust in immunisation programme/immunisation is harmful to the child's health	All	Negative
		
Fear of vaccine side effects leading to drop out or delayed immunisation	Mostly female FGDs	Negative
		
***Programmatic preferences***		
Preference for routine immunisation services	All	Positive
***Reasons for preference of routine services:***		
Health workers take responsibility if complications develop after immunisation	All	Positive
Routine services have a fixed address	All	Positive
Forceful methods of conducting mass immunisation exercises	All	Negative

### Social influence

Supportive kin recognising the benefits of immunisation were essential to the child being immunised. In this study setting the decision to go for immunisation was generally a joint decision between the mother and father of the child. Most study participants (FGDs and KIIs) strongly emphasized that both the child's mother and father were responsible for the immunisation of the child, but in reality, only women were in charge of taking children for immunisation. Below, we present the influences on the mother's behaviour from her social context, first her male partner, second the older generation such as mothers-in-law, fathers-in-law, sisters-in-law, and lastly her peers.

Most women expressed support from their partners when taking the child for immunisation, such as money for transport and a granted permission to take the child for immunisation. This support was reiterated by male participants; both KII and FGD participants.

*'As for me, I make sure that when my wife is pregnant she attends the antenatal clinic as required and is also immunised because she usually tells me when she is immunised. Also after she gives birth I make sure she takes the children for immunisation on the dates written on the immunisation card.' *(FGD with fathers)

A minority of male participants rejected immunisation however and therefore hindered their wives from immunising their children.

*'My wife is pregnant but she has not been immunised. She has a four year old child and she talks about immunising the child but I stop her from doing it. For me I don't believe in it. As you can see I am a mature person but I did not grow up because of that *(immunisation). *It was better for me to use traditional medicine to treat fever for example, but because these days the fever is very strong I now use tablets *(for treatment). *Even these injections *(from immunisation) *paralyze people I know, and we also see them in books and in pictures.' *(FGD with fathers)

If a father disagreed to immunisation, the mothers expressed less power for decision making at the household level which made them unable to take their children for immunisation. They said that it was the man's prerogative to make the decision to immunise the child. So if the father of the child stopped them from taking children for immunisation then they would not immunise their children. The women who felt this way were mainly young and with lower level of education. They believed they should submit to the men's directives at all times.

*'Because the wife fears the husband, if you give me instructions never to leave the home, can I leave it? I will have broken a rule.' *(FGD with younger mothers)

If a child fell sick it would be the mothers who would spend 'sleepless nights' while the men slept or went to work. Some women, mostly in FGDs with older mothers, therefore made decisions to immunise their children despite opposition and threats from their husbands, and they stressed that this has to be done with determination.

*'Also, it is the mother who should really make sure your child is immunised. If you follow the man's advice and you don't immunise your child, when that child falls sick it is you the mother who will spend sleepless nights when the child is sick. He will be snoring and the doctors will abuse you as he is not around the hospital. Yet you followed his advice. You the mother have to stick to your guns. Let him fight with you, but after your child has been immunised.' *(FGD with older mothers)

Some women who opposed their husbands' decision not to immunise reported that they had to be discreet about the whole process of immunisation. One female key informant told how she pretended she was going to the market, but went to the nearby outreach centre for immunisation. This 'rebellious' behaviour had consequences such as intimate partner violence which included emotional, verbal and physical violence. The violence after immunisation was experienced by a minority of women both in the FGDs and KIIs. However, all female participants in the FGDs and KIIs reported it since they had witnessed or heard about this occurrence. An older mother in tears reported an incident after her child got the first injection on the thigh, '*the baby cried all night'*. Her husband sent her and the baby out of the house in the night saying it was her decision to immunise the child and he could not tolerate the noise.

Even amidst spousal violence some women derived satisfaction from the fact that their children were immunised particularly the older women that had disobeyed the husband's instructions and they intended to take their children for subsequent immunisations.

Only women who were convinced about the benefits of immunisation were willing to endure the consequences of opposing their spouses.

*'The mother will say, "Let me immunise my children for their good because when my child is disabled, my husband can have other children with another woman. It is me to suffer with my children who would have helped me in future." *(Female key informant)

It was reported by participants in FGDs and KIIs that if the father was against immunisation it was also common to be influenced from his elder relatives and personal experiences.

*'Like I explained before about some elderly women who claim children will become lame after immunisation, some men use that excuse because they had ever heard of it while still young. So when they grow up and get children they say the children will become lame or get brain damage. That is why you see some children when they get measles they almost die because the husband refused the wife to take children for immunisation.' *(FGD with older mothers)

The men who disagreed to immunisation were put under pressure by his elderly relatives and they had strong union.

*'Like for the old people who have previously heard that children died, when you tell them that you are taking a child for immunisation, they will not like it. No, his father will tell him that why did you let her take the child for immunisation. And he will answer that I refused her but she insisted.' *(FGD with older mothers)

The older generation exerted influence on the mother's behaviour indirectly through the husband as shown above, and also directly. For instance, the younger mothers were persuaded to take their children for immunisation against spousal consent by older experienced women in their neighbourhood. They were supported in breaking some household rules to protect the wellbeing of their children. However, older women were sometimes not supportive of young women. This was highlighted by one of the female key informants in charge of community mobilisation. She reported that teenage mothers were stigmatized by the older women who laughed at them for giving birth at an 'early age'. In addition some teenage mothers were told by the older women in their community that the fathers of their children were HIV positive. These younger mothers therefore stayed away from all immunisation activities where it was possible to meet these older women from whom they faced social stigma.

Female participants felt that mostly men had a non-conforming attitude towards childhood immunisation. So they described these men as having '*weak brains'*, lazy, and irresponsible. This was also reflected among male participant's judgement against those that did not immunise children: terms like they are *'ignorant' *or *'uneducated' *were used to describe them. The female respondents especially felt that this non-conforming attitude should have consequences. They stressed that individuals in this category should be given some form of punishment by the government. Some male respondents were in agreement with the women because they reasoned that these individuals were cruel to the innocent children whose future they were *'sabotaging' *or *'ruining' *by refusing immunisation. Other men strongly opposed the idea of punishment however arguing that it would be difficult to identify such individuals in the community.

In general, both FGDs and KIIs supported that the mother was under strong social influence affecting decision making on immunisation.

### Self -efficacy

Not only the mother's social context influenced immunisation behaviour, but also her own ability to overcome barriers, defined as self-efficacy, affected behaviour. Major hindrances reported included financial deprivation which made the cost of going for immunisation a considerable decision to make.

*'If I don't have food, how can I use Uganda shillings 2000 (approximately US$1) for a boda-boda *(means of transport using motorcycle/bicycle) *to go for immunisation?' *(FGD with younger mothers)

With lack of money, walking could be the only alternative with distances of up to 4 km and having to cross two motor highways in some instances. This was reported from all FGD participants as a major challenge especially for women in the post-partum period if they needed to take their children for immunisation. This challenge was compounded by frequent reports by mothers and fathers that they were not given the anticipated services due to vaccines being out of stock or due to absent health workers.

Another expense for the mothers was not only financial, but also related to time. Vaccination could easily take one day and they would have lost the potential income for that day.

*'The nature of work for some people at times makes them miss these immunisation schedules since somebody leaves home at around 6.00 a.m. and comes back at around 6.00 p.m. During the day this person is at the stone quarry *(work place) *about 2 kms from here, now the person will not take the child for immunisation although the person will be willing to take the child.' *(FGD with fathers)

It was reported from Female FGDs especially that poor mothers often felt stigmatised and bullied from other women and health workers if they did not show up in good clothing or with presentable clothes or shawl for their children.

*'Some young women fear going for immunisation because they don't have a baby shawl for carrying the children to hospital so when you reach at the hospital with some sheets which are not clean some nurses will sometimes begin abusing you.' *(FGD with older mothers)	

Gender roles were perceived as a barrier to male involvement in child immunisation activities and it was hard for the men to overcome these barriers. They would 'feel out of place' at the immunisation centres as it was considered a 'female arena.' Even if the men were willing to take their children for immunisation they did not have time to do this because they had to go for work. This competing demand for time was emphasised in all FGDs and supported by the KIIs as an important barrier to immunisation activities. Lack of job security and high unemployment rates forced parents to serve their employer if they were on private ad-hoc or longer contracts at the expense of personal activities such as taking the child for immunisation.

### Attitudinal factors

The convictions of the respondents towards childhood immunisation were classified into three sub-themes: 1) trust in immunisation 2) fear of vaccine side effects 3) programmatic preferences.

#### Trust in immunisation

There were two opposing beliefs among our study participants: those who trusted in immunisation as a child survival strategy and those who feared or refused immunisation. Those who trusted vaccines were generally better educated and older. They recognised the diseases that could cause severe outcomes in children such as physical disability or death. These diseases were perceived by most FGD participants and key informants as common and the children as vulnerable to get the diseases unless immunised.

*'When the child gets measles, he will not be bedridden. He will just get a rash or cough. He may also get red eyes or mouth rash but he will be able to play as usual. But if he was not immunised he will get very high temperatures, fever, diaorrhea and you become worried the child may even die.' *(FGD with younger mothers)

The societal value of having a healthy child population was strongly held among most study participants. If their children survived vaccine preventable diseases they could contribute to building a strong society and become *'doctors and teachers who would be able to treat or teach the population.'*

The fear of perceived ill effects of immunisation underpinned the strong belief against immunisation. All study participants perceived that a lack of trust towards vaccines existed among community members. Common beliefs were that vaccines were '*expired*' and could cause '*physical disability and/or death' *among their children. The perceived susceptibility of their children to suffer from severe effects of the vaccines led some to decline immunisation.

*'At one time our neighbour in *'rural geographical area' *immunised a child in the morning and by 5.00 p.m. the child was dead. From that time I fear taking children for immunisation and all my children are not immunised.' *(FGD with fathers)

A lack of trust was also observed against the health personnel believed not to check the drugs properly and only give *'expired' *vaccines which might cause disability or death.

#### Vaccine side effects

Among those who accepted the benefits of immunisation, side effects were recognised as a constraint. This fear of vaccine side effects was more commonly held among female than male respondents. Many had experienced or were afraid of vaccine side effects such as fever, temporary *'paralysis of the leg' *and excessive crying after the *'first injection given on the thigh'*. The consequences were either declining or delaying subsequent immunisations.

*'Sometimes after immunisation children get fever and spend the whole night crying so the health worker must tell the mother in advance what will happen to the baby, "that the baby might become weak, or get a fever or the injection is painful so he will cry a lot." But some health workers don't warn the parents so when they reach home the mother will notice the child has got a fever or is crying uncontrollably. And this makes her worried.' *(Male key informant)

Home remedies against side effects were frequently reported. Some treated the child with paracetamol or 'junior aspirin.' However many female FGD participants said that the side effects were persistent even after drugs were tried out, so they applied ice, cold water, onions, oranges, or 'black' shoe polish at the injection site. They explained that when the child was injected on the thigh, the vaccine remained *'stationary' *at the injection site and that is why the child suffered. When applying the "black shoe polish" or any other home remedy the vaccine *'was moved' *(absorbed) and the child got some relief.

#### Programmatic preferences

Routine immunisation was distinguished from mass immunisation services. Routine immunisation services involve individually scheduled immunisations according to the expanded programme for immunisation. Caretakers then have to bring the child to the immunisation units. On the other hand mass immunisation is the distribution of one particular or a group of vaccines meant for everybody within a certain age range in the area. It is meant to compensate for not achieving full immunisation coverage by including those who have got and those who would have missed the routine immunisation. Mass immunisation activities mostly promote measles as well as polio vaccination among children under five years and are done as community outreaches, in schools, and in conjunction with religious groups like churches. Among study respondents that accepted the benefits of immunisation, most preferred routine compared to mass immunisations because they believed that routine immunisation was *'safe'*. Several reasons were fronted for preferring routine services: first, if the child developed complications after immunisation it would be easier to trace the health workers conducting routine services, which was not the case in mass immunisation services. The health worker could *'take responsibility' *during routine services compared to mass immunisation services which lacked a *'fixed physical address.' *Second, it was difficult for the parents to understand that there was need to take their children for further immunisations when they had completed the routine immunisation schedule. Only a few study participants had voluntarily taken their children for mass immunisation activities. The misgivings towards mass immunisation were worsened by the reported methods used to conduct the mass immunisation including officials forcefully immunising the children.

## Discussion

Significant others particularly the male partner played an important role on the mother's decision making that could be prohibitive for immunisation. A few of the husbands held an opposing view to immunisation and stopped their wives from immunising their children. Lack of clothing, money for transport, or time influenced whether parents took their children for immunisation or not. Most respondents expressed their trust in the benefits of immunisation but had reservations towards the associated side effects. Others expressed a total distrust of immunisation programmes and vaccines. Those with less education showed more scepticism towards immunisation. Study respondents also preferred routine to mass immunisation activities. Our findings create deeper understanding and complement quantitative studies highlighting associations between immunisation behaviour and maternal education, wealth, age and parity [3,4,6-9].

In our study, husbands and other kin sometimes hindered the mother from immunising her child. A qualitative study from Hong Kong reported that most participants did not receive advice from family members although siblings and peers encouraged participants to take more immunisations than what was provided in the EPI programme [5]. The difference in study findings from our study could be due to better access to technology and health information, and due to mandatory immunisation for school entry in Hong Kong. In developing countries such as Uganda, decision making in health seeking behaviour is a very complex process, and earlier studies strongly emphasize the social dimensions [13]. Newer research focusing on the influence of the husband on the mother's health behaviour show that existing organisation of services and lack of contextualisation in health programmes causes reduced male involvement. Lack of male involvement is prohibitive for successful maternal and child health programmes [27-29].

Uganda as a patrilineal society directly increases the man's power to inhibit child immunisation, for instance, he can stop the mother from spending money on transport for immunisation. In our study, the younger mothers particularly reported being disempowered economically and at decision making at the household level sometimes resulting into intimate partner violence. This supports the literature on widespread intimate partner violence in Ugandan communities [30,31]. The violence is often a result of disturbing social relations. Social relations are particularly affected when illness or death occurs, and quarrels and blaming may arise placing guilt on partners. Thus, a fear of disturbing the social equilibrium greatly influences the health decision making processes [13]. Yet the health system has traditionally targeted mothers because they spend more time with the children and are caretakers of the sick [21,22]. It is important therefore to carefully consider the social contexts during programme design and implementation for child immunisation.

Respondents in our study expressed fear of child death or physical disability and these were categorised into two: for and against immunisation. Either category was motivated by a preference for a state free of illness [13]. Those that were against immunisation wanted a state free of disability or illness from the vaccines. Those that believed in immunisation wanted a state free of illness caused by the vaccine preventable diseases. In the Hong Kong study mentioned earlier, participants' decision to immunise their children was often the result of weighing the benefits against the risks and most felt that the benefits outweighed the risks [5]. Another study showed that personal experiences, value systems and level of trust in health professionals were fundamental to parental decision making about immunisation even when challenged by anti-immunisation messages [10]. The ones that cause most concern are those that are against immunisation due to perceived health risks from vaccines. Their concerns should be addressed systematically by the health workers since inefficient response to public debates about vaccines has the potential to be amplified and to significantly reduce immunisation coverage as was seen in the case of measles-mumps-rubella vaccine and autism [32].

Even among study participants that accepted the benefits of immunisation in our study, side effects were recognised as a constraint. The reported behaviour of the mothers after experiencing side effects indicates that the health workers need to strengthen the strategy of increasing immunisation coverage. High levels of immunisation can be achieved through use of information, education, and persuasion on immunisation concepts and the rationale for immunisation rather than use only images of children suffering from vaccine preventable diseases or historic images relating to outbreaks [32].

Behavioural or social science theories provide the basis for understanding health behaviour. Several models have been utilized in this sense; such as the health belief model, theory of planned behaviour, the theory of reasoned action, the attitude-social influence-self-efficacy (ASE) model. Although the ASE model resembles the theory of planned behaviour, it has evolved as a separate model, with a different methodological nature [15,16]. The ASE model was better suited and provided a useful framework to analyse the factors associated with immunisation behaviour in our study because it addresses self efficacy. Self efficacy is better suited to dyadic behaviour such as taking the child for immunisation than volitional control which is assumed by the theory of reasoned action.

### Methodological considerations

In this study triangulation was achieved by using different study methods and respondents, and by having researchers from different backgrounds, from social science and medicine. Although qualitative methods do not give the magnitude and variations across the different categories of the respondents, the triangulation in this study aimed at reaching an objective view of the data and was useful to check the consistency and contradictions across and within groups [26,33]. However, the following study limitations need to be considered in the interpretation of these results: first, there was a potential for the local council leaders to omit eligible respondents and to choose individuals that were close to them. This was mitigated by explaining the criteria for study inclusion to the leaders. These leaders mobilised potential participants which tended to be large groups of participants of up to 25 study participants. The research team together with the local leader then selected FGD participants from this large group. Second, JNB's presence (a medical doctor) could have led to eliciting socially desirable answers in the discussions. This was mitigated by assuring the respondents of confidentiality. However, participants' criticisms of immunisation indicate that the potential bias arising from this limitation was greatly reduced. Third, the use of a translator for one of the FGDs may have led to loss of depth of the issues being discussed. However the similarity of the themes with the other FGDs is testimony that the key findings were similar. In addition, JNB who was present at this FGD asked whether key issues were being probed. Fourth, there was a tendency in this study setting for participants to sometimes speak about their experiences in the third person. Therefore it was sometimes difficult to decipher whether individuals were speaking about themselves or other people. This was mitigated during data analysis through taking quotes within their context using an FGD or KII as a unit of analysis. In addition, the quotes presented here were representative of ideas which were common across data sources and in other settings these would be presented in the first person.

## Conclusions

The non-supportive role of husbands played an important role on the mother's decision making that could hinder utilisation of immunisation. In addition, gender roles in this setting were prohibitive for male involvement in immunisation services. Lack of clothing, money for transport or competition between work and child immunisation influenced whether parents took their children for immunisation or not. Distrust of immunisation programmes and vaccines was expressed. Study respondents also preferred routine to mass immunisation activities. Immunisation programmes in developing countries have traditionally employed interventions that target mothers to improve immunisation coverage. These interventions need to be re-designed in order to address socio-cultural contexts, involve the community and not only target mothers for childhood immunisation. There is need for a multidisciplinary community programme that empowers women economically and in decision making at the household level, and helps men recognise the role of women in decision making for child health. Further research identifying effective and innovative strategies for increasing male involvement in immunisation services in this setting is essential. A general need for increased understanding on management of vaccine side effects, the rationale for immunisation and mass immunisation were observed in this study. Health education aiming at informing rather than creating fear of vaccine preventable diseases could empower parents to make informed decisions and would increase male support for their partners/spouses. Health workers require additional in-service training on medical ethics to promote the culture of treating people with dignity and respect. In addition, effective communication skills should be strengthened to address the various issues which could potentially reduce immunisation coverage such as health worker's response to mother's appearance, the fear of vaccines and vaccine side effects.

## Competing interests

The authors declare that they have no competing interests.

## Authors' contributions

JNB, IMSE contributed to the conception and design of the study, data analysis, interpretation of data and in drafting the paper. FN contributed to the conception and design of the study; interpretation of data; and in drafting the paper. ER, JK contributed to data analysis, interpretation of data and in drafting the paper. HW contributed to interpretation and drafting of the manuscript. All authors approved the final manuscript.

## Pre-publication history

The pre-publication history for this paper can be accessed here:

http://www.biomedcentral.com/1471-2458/11/723/prepub
